# Supplementary material for: Assessing diversity in multiplex networks
Source: Sci Rep. 2019 Mar 14;9:4511. doi: 10.1038/s41598-019-38869-0 (PMC6418208; doi:10.1038/s41598-019-38869-0)
Supplement: Supplementary file 1 — Supporting Information Material [file 41598_2019_38869_MOESM1_ESM.pdf]

Supporting information material: Assessing diversity in multiplex networks

Laura C. Carpi,[1] Tiago A. Schieber,[2] Panos M. Pardalos,[3] Gemma Marfany,[4,5]
Cristina Masoller,[6] Albert Díaz-Guilera,[7,8] and Martín G. Ravetti[9,*]

[1]*Programa de Pós-Graduação em Modelagem Matemática e Computacional, PPGMMC, Centro Federal de Educação Tecnológica de Minas Gerais, CEFET-MG. Av. Amazonas, 7675. 30510-000. Belo Horizonte, MG, Brazil*
[2]*Departmento de Ciências Administrativas, Universidade Federal de Minas Gerais, Belo Horizonte, MG, Brazil*
[3]*Industrial and Systems Engineering, University of Florida, Gainesville, FL, USA*
[4]*Departament de Genètica, Microbiologia i Estadística, Facultat de Biologia, Universitat de Barcelona, Barcelona, Spain*
[5]*Institut de Biomedicina de la Universitat de Barcelona (IBUB-IRSJD), Barcelona, Spain*
[6]*Departament de Física, Universitat Politècnica de Catalunya. Rambla St. Nebridi 22, Terrassa 08222, Barcelona, Spain*
[7]*Departament de Física Fonamental, Universitat de Barcelona, Barcelona, Spain*
[8]*Universitat de Barcelona, Institute of Complex Systems (UBICS), 08028 Barcelona, Spain*
[9]*Departmento de Engenharia de Produção, Universidade Federal de Minas Gerais, Belo Horizonte, MG, Brazil*

(Dated: December 3, 2018)

NOTE S1. PROOF THAT D IS A METRIC BETWEEN NETWORKS

Equation (1) from the main text shows that $D(p,q) = 0$ if, and only if, both networks possess the same transition matrix and, consequently, the same adjacency matrix. D is a metric because the Jensen-Shannon divergence is square of a metric between probability distributions, then $\mathcal{D}$ is a metric between layers, in fact, is a metric between labelled graphs.

Figure S1 and Table S1 presents a small example on how the metric works. Both networks are very similar, they have the same number of nodes, and all of them have the same degree. As it can be seen in Table S1, nodes 1 and 4 present dissimilarity zero. Node 1 has the same adjacency matrix in both networks and it is connected to different nodes at distance 2, then, node 1 has the same node distance distribution in both networks. The same is valid for node 4 that it is connected to nodes 1 and 2 in both layers. In this small example it is easy to see that the distance between networks is zero if all nodes share, with their counterparts in the other layers, the same adjacency matrix.

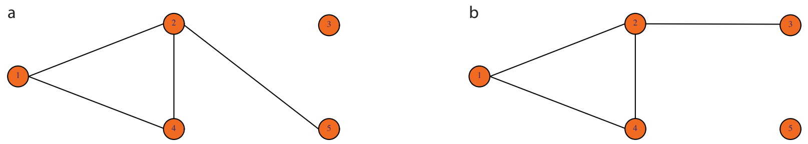

FIG. S1: **Nodes and layer difference metrics.** Example of the node and layer difference metrics in a bilayer network. Nodes and layers difference values are presented in Table S1.

TABLE S1: Nodes and layer difference values for networks depicted in Figure S1.

Nodes	$\mathcal{D}_i(a,b)$
1	0
2	0.2409
3	0.7247
4	0
5	0.7247
$\mathcal{D}(a,b)$	0.4059

NOTE S2. OTHER MEASUREMENTS

Here we discuss and compare different existing measures and methods that are used either, to compute dissimilarities between labeled nodes, or heterogeneity in multiplex structure. Table S2 presents, to the best of our knowledge, the most commonly used methods.

TABLE S2: Methods used for comparing multiplex structures.

**Measure**	**Description**	**References**
Graph Edit Distance (GED)	Counts only the number of uncommon edges between two networks, not considering topological differences between them.	[1–3]
The Quantum Jensen-Shannon divergence (QJSD)	It is not proved to be a metric between networks. It is computed through the square root of the Jensen-Shannon divergence between the eigenvalues of the normalized Laplacian Matrix. The main drawbacks of this measure are, the lack of local information and the number of isospectral networks with different topological features.	[4–6]
Node and Layer activity vector	The node-activity value is a binary operator returning 1 if the node possesses at least one first neighbor. The layer-activity vector is a vector containing all node activity value of the layer. In order to quantify the relative overlap between two layers at the level of node activity, Hamming distance between the two corresponding layer-activity vectors was proposed in [7]. Since it returns zero if the networks share the same set of active nodes, is a pseudometric between networks. Therefore, pairs of connected networks are indiscernible using this measure.	[7, 8]
Interlayer Mutual Information	Computes how correlated the degree distributions of a pair of layers are. The main drawback is the lack of information when networks with the same degree distribution, but different topological structure, are compared. For instance, a pair of networks can possess a high interlayer mutual information value, not possessing common links.	[9]
Average Edge Overlap	Global measure of the multiplex system which computes the expected number of layers on which an edge is present. The main drawback is the lack of information concerning local and global features of the system.	[9]

To highlight the fact that our measure looks beyond the degree distribution, we compare a Barabàsi-Albert (BA) scale-free network (m=2), and two networks generated by dk model [10], with $k = 1$ preserving its degree sequence and $k = 2.5$ preserving the degree sequence, degree correlation, clustering coefficient and clustering spectrum. We compute the node dissimilarity $\mathcal{D}_i$ corresponding to the node with the highest degree in the BA network, and the layer dissimilarities, as shown in Figure S2. It is possible to see that, although each corresponding node in these networks has the same degree, $\mathcal{D}$ recognizes that nodes are connected in a different way, giving different dissimilarity values. Measures based on the node degree, or on node activity, are no able to acknowledge this fact.

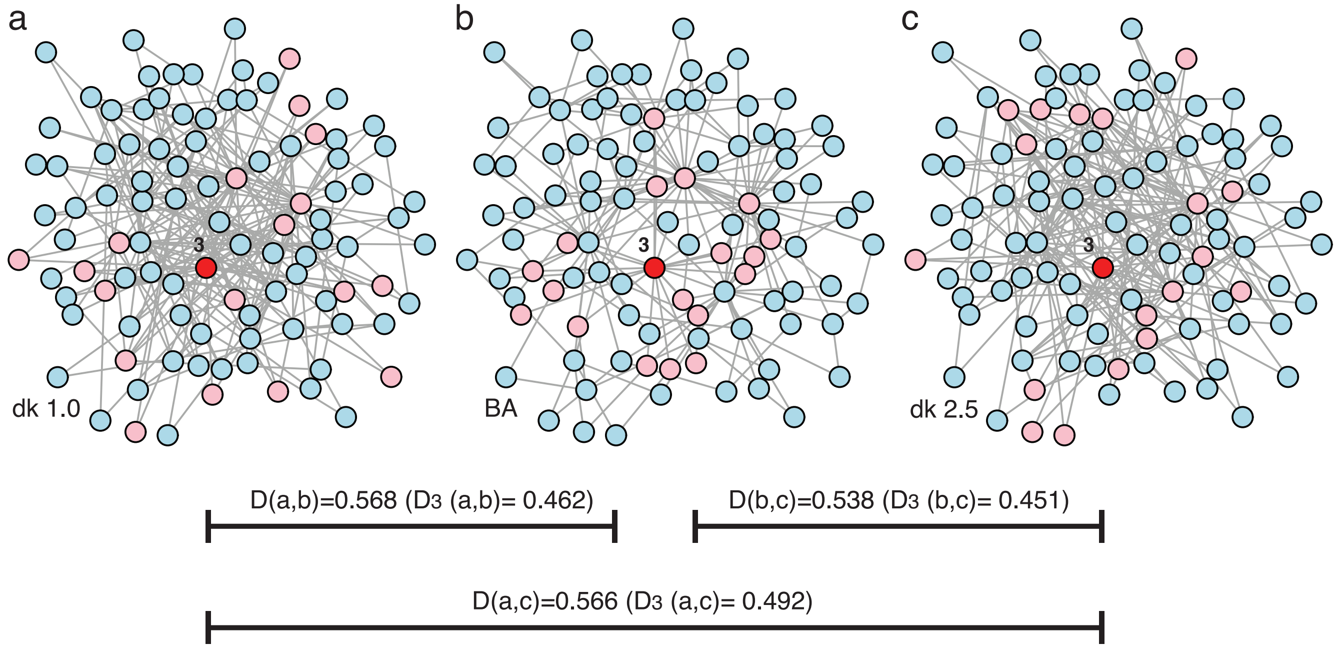

FIG. S2: **Dk model experiment**. Layer dissimilarity values for a Barabàsi-Albert (BA) scale-free network and two different networks generated through the dK null model. (b) BA network (N=100, m=2). (a) Network dk 1.0 preserves the BA degree sequence. (c) Network dk 2.5 preserves the joint degree distribution and clustering spectrum. Red colored node (ID=3) corresponds to a node with degree 18, and pink nodes are the ones connected to it. $\mathcal{D}_3$ correspond to the node dissimilarity values of node 3 in the different layers.

In a previous work, our group proposed a pseudo-metric between graphs, a measure that is not designed to consider the identity of the nodes and to whom they are connected [11]. Then, this previously proposed measure cannot be applied to structures in which the position of specific nodes and their relationship with all other in the network is relevant. Some examples where labels are relevant are, for example, climate networks where each node is connected to the others depending on the variable considered, or social networks in which the same group of individuals is connected considering different social ties.

To illustrate the limitation of the measure presented in [11]when applied to multiplex networks, we present Figure S3, in which, nodes 1, 2 and 3 are connected in different ways by two links. As the distance proposed in [11] does not consider the identity of the nodes, networks A and B are seen as identical ($D = 0$). The measure developed in this work considers the identity of the nodes and captures the topological differences between networks A and B ($D = 0.3109697$).

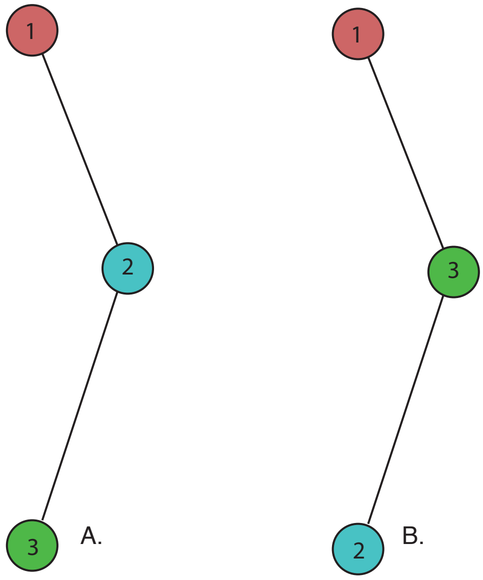

FIG. S3: The distance defined in [1] does not capture the changes between labels 1 and 2 in networks A and B, then the structures are seen as equals (D=0). The new measure on the other hand, is capable of capturing and quantifying the difference (D=0.3109697).

NOTE S3. EXPERIMENT ON AARHUS MULTIPLEX NETWORK STRUCTURE.

Layers difference values

Matrix S2 presents the difference values between layers.

$$\mathcal{D}(p,q) = \begin{bmatrix} - & \text{lunch} & \text{facebook} & \text{coauthor} & \text{leisure} & \text{work} \\ \text{lunch} & 0 & 0.8273 & 0.9251 & 0.6731 & 0.5913 \\ \text{facebook} & 0.8273 & 0 & 0.4973 & 0.6073 & 0.8027 \\ \text{coauthor} & 0.9251 & 0.4973 & 0 & 0.6315 & 0.9052 \\ \text{leisure} & 0.6731 & 0.6073 & 0.6315 & 0 & 0.7129 \\ \text{work} & 0.5913 & 0.8027 & 0.9052 & 0.7129 & 0 \end{bmatrix} \tag{S2}$$

Node difference values

For the two highest and lowest values of node diversity, nodes 1,11, 58 and 60, we present their difference matrices, and local diversity values.

Difference matrix of node 1, Matrix S3. Its local diversity value is U_1=0.9844.

$$\mathcal{D}_1(p,q) = \begin{bmatrix} - & \text{lunch} & \text{facebook} & \text{coauthor} & \text{leisure} & \text{work} \\ \text{lunch} & 0 & 0.9844 & 0.9844 & 0.9844 & 0.9844 \\ \text{facebook} & 0.9844 & 0 & 0 & 0 & 0 \\ \text{co-authorship} & 0.9844 & 0 & 0 & 0 & 0 \\ \text{leisure} & 0.9844 & 0 & 0 & 0 & 0 \\ \text{work} & 0.9844 & 0 & 0 & 0 & 0 \end{bmatrix} \tag{S3}$$

Difference matrix of node 11, Matrix S4. Its local diversity value is U_{11}=3.1163.

$$\mathcal{D}_{11}(p,q) = \begin{bmatrix} - & \text{lunch} & \text{facebook} & \text{coauthor} & \text{leisure} & \text{work} \\ \text{lunch} & 0 & 0.9844 & 0.8801 & 0.752 & 0.7366 \\ \text{facebook} & 0.9844 & 0 & 0.5458 & 0.8575 & 0.9844 \\ \text{co-authorship} & 0.8801 & 0.5458 & 0 & 0.8527 & 0.9276 \\ \text{leisure} & 0.752 & 0.8575 & 0.8527 & 0 & 0.8495 \\ \text{work} & 0.7366 & 0.9844 & 0.9276 & 0.8495 & 0 \end{bmatrix} \tag{S4}$$

Difference matrix of node 58, Matrix S5. Its local diversity value is U_{58}=3.0643.

$$\mathcal{D}_{58}(p,q) = \begin{bmatrix} - & \text{lunch} & \text{facebook} & \text{coauthor} & \text{leisure} & \text{work} \\ \text{lunch} & 0 & 0.9844 & 0.8801 & 0.752 & 0.7366 \\ \text{facebook} & 0.9844 & 0 & 0.5458 & 0.8575 & 0.9844 \\ \text{co-authorship} & 0.8801 & 0.5458 & 0 & 0.8527 & 0.9276 \\ \text{leisure} & 0.752 & 0.8575 & 0.8527 & 0 & 0.8495 \\ \text{work} & 0.7366 & 0.9844 & 0.9276 & 0.8495 & 0 \end{bmatrix} \tag{S5}$$

Difference matrix of node 60, Matrix S6. Its local diversity value is U_{60}=0.9844.

$$\mathcal{D}_{60}(p,q) = \begin{bmatrix} - & \text{lunch} & \text{facebook} & \text{coauthor} & \text{leisure} & \text{work} \\ \text{lunch} & 0 & 0 & 0 & 0 & 0.9844 \\ \text{facebook} & 0 & 0 & 0 & 0 & 0.9844 \\ \text{co-authorship} & 0 & 0 & 0 & 0 & 0.9844 \\ \text{leisure} & 0 & 0 & 0 & 0 & 0.9844 \\ \text{work} & 0.9844 & 0.9844 & 0.9844 & 0.9844 & 0 \end{bmatrix} \tag{S6}$$

NOTE S4. HIV-1 MULTIPLEX NETWORK.

The complete dataset is available as Supporting Information material in an Excel file at [12]. In the same file, the reader will find the diversity value for each on of the 1114 genes.

tat is an essential regulatory element. It is a HIV trans-activator and plays an important role in regulating the transcription of the viral genome [13–17].

nef and *vif* are considered belonging to the class of accessory regulatory proteins. *nef* is involved in multiple functions during the replication cycle of the virus, playing an important role to increase virus infectivity. *vif* is important for the infectivity of HIV-1 virions depending on the cell type [13–17].

The *env* and *gag* genes belongs to the class of viral structural proteins. *gag* codes for the precursor gag-polyprotein which is processed by viral protease during maturation of the protein matrix and *env* is responsible for a mechanism that embeds in the viral envelope to enable the virus to attach to and fuse with target cells [13–17].

NOTE S5. EUROPEAN AIR TRANSPORTATION NETWORK.

TABLE S3: Airlines considered in the experiments, the X indicates the participation of the airline in one of the three alliances.

	Airlines	One World	Star Aliance	Skyteam
1	Lufthansa		X	
2	Ryanair			
3	Easyjet			
4	British Airways	X		
5	Turkish Airlines		X	
6	Air Berlin	X		
7	Air France			X
8	Scandinavian Airlines		X	
9	KLM			X
10	Alitalia			X
11	Swiss International Air Lines		X	
12	Iberia	X		
13	Norwegian Air Shuttle			
14	Austrian Airlines		X	
15	Flybe			
16	Wizz Air			
17	TAP Portugal		X	
18	Brussels Airlines		X	
19	Finnair	X		
20	LOT Polish Airlines		X	
21	Vueling Airlines			
22	Air Nostrum			
23	Air Lingus			
24	Germanwings			
25	Pegasus Airlines			
26	Netjets			
27	Transavia Holland			
28	Niki			
29	SunExpress			
30	Aegean Airlines		X	
31	Czech Airlines			X
32	European Air Transport			
33	Malev Hungarian Airlines			
34	Air Baltic			
35	Wideroe			
36	TNT Airways			
37	Olympic Air			

Difference matrix of the Star Alliance network.

$\mathcal{D}(p,q) =$

–	Lufthansa	Turkish	Scandinavian	Swiss	Austrian	TAP	Brussels	Polish	Aegean
Lufthansa	0	0.197	0.1906	0.1548	0.1663	0.1659	0.1564	0.1572	0.1809
Turkish	0.197	0	0.1739	0.13	0.1481	0.1421	0.1435	0.1402	0.1502
Scandinavian	0.1906	0.1739	0	0.1179	0.1453	0.1169	0.1202	0.1164	0.125
Swiss	0.1548	0.13	0.1179	0	0.1071	0.0824	0.0811	0.084	0.0904
Austrian	0.1663	0.1481	0.1453	0.1071	0	0.1145	0.1143	0.1075	0.1163
TAP	0.1659	0.1421	0.1169	0.0824	0.1145	0	0.0779	0.0872	0.0917
Brussels	0.1564	0.1435	0.1202	0.0811	0.1143	0.0779	0	0.0877	0.0905
Polish	0.1572	0.1402	0.1164	0.084	0.1075	0.0872	0.0877	0	0.0881
Aegean	0.1809	0.1502	0.125	0.0904	0.1163	0.0917	0.0905	0.0881	0

Pierre Auger Collaboration

Global Diversity Value (1.65795)	
10 Most Diverse Nodes	Node Diversity Value
4	3.889323
204	3.780326
45	3.262540
211	3.162804
54	3.122001
125	3.043063
1	3.043063
53	3.011467
73	2.806901
57	2.794919

TABLE S4: Pierre Auger Collaboration: global and node diversity values of the 10 most diverse nodes in the multiplex network.

Homo Sapiens

Global Diversity Value (1.692839)	
10 Most Diverse Nodes	Node Diversity Value
MCM6	4.008031
SUMO2	3.958065
CDK2	3.957474
PSMA5	3.952747
PSMD1	3.951968
PAIP2	3.945939
BMP7	3.943887
PSMA4	3.940710
CREB1	3.939096
HDAC2	3.933005

TABLE S5: Human Genome: global and node diversity values of the 10 most diverse nodes in the multiplex network.

NOTE S6. QUANTIFICATION OF DIVERSITY IN OTHER REAL NETWORKS.

Pierre Auger Collaboration: the network consists of layers corresponding to different working tasks within the Pierre Auger Collaboration. considering all submissions between 2010 and 2012 and assigned each report to L=16 layers according to its keywords and its content: Neutrinos, Detector, Enhancements, Anisotropy, Point-source, Mass-composition, Horizontal, Hybrid-reconstruction, Spectrum, Photons, Atmospheric, SD-reconstruction, Hadronic-interactions, Exotics, Magnetic and Astrophysical-scenarios. Readers should refer to [18] for details. The multiplex is weighted (see Table S4).

Homo Sapiens - genetic interaction: network concerns homo sapiens genetic interaction. There are 18222 nodes and 7 layers: Direct interaction, Physical association, Suppressive genetic interaction defined by inequality, Association, Colocalization, Additive genetic interaction defined by inequality and Synthetic genetic interaction defined by inequality. See [19, 20] for a better description of the data and Table S5 for the results.

Hepatitusc multiplex GPI network: is the multiplex genetic and protein interactions network of the Hepatitus C virus. The network contains 105 nodes, and 3 layers: Physical association, Direct interaction and Colocalization. Readers should refer to [19, 20] for a better description of the data and Table S6 for the results.

Human-Herpes4 multiplex GPI network: representing the multiplex genetic and protein interactions network of the EpsteinBarr virus, also known as human herpes-virus 4 (HHV-4). The network contains 216 nodes, and 4 layers: Physical association, Direct interaction, Association and Colocalization. Readers should refer to [19, 20] for a better description of the data and Table S7 for the results.

NYclimatemarch2014 multiplex social network: represents different types of social relationships amoung users, obtained from Twitter during the People's Climate March in 2014. The multiplex network used in the paper makes use of 3 layers, corresponding to retweet, mentions and replies observed between 2014-09-19 at 00:46:19 to 2014-09-22 at 06:56:25. There are 102439 nodes, labelled with integer ID between 1 and 102439. The multiplex is weighted

Hepatitus C virus

Global Diversity Value (1.002419)	
10 Most Diverse Nodes	Node Diversity Value
HCVgp1	1.38941
SMURF2	1.02580
SMURF1	1.02580
SMAD3	1.02580
NAP1L1	1.02580
PSMB9	1.02580
EFEMP1	1.02580
MOB1A	1.02580
FKBP8	1.02580
TP53	1.02580

TABLE S6: Human Genome: global and node diversity values of the 10 most diverse nodes in the Hepatitus C virus multiplex network.

Herpes virus 4

Global Diversity Value (0.8776402)	
10 Most Diverse Nodes	Node Diversity Value
EBNA-LP	1.739951
DDX5	1.463567
EBNA-3B/EBNA-3C	1.406072
EBNA-1	1.273689
BAG2	1.211084
HSPA4	1.211084
CDKN2A	1.206365
TUBB	1.184846
TUBA1B	1.184846
HSPA8	1.184846

TABLE S7: Human Genome: global and node diversity values of the 10 most diverse nodes in the Herpes virus 4 multiplex network.

(obtained by summing up the number of a specific type of interaction over time).
London Transportation Network: Nodes are train stations in London and edges encode existing routes between stations. Underground, Overground and DLR stations are considered. There are 369 nodes in total. Readers should refer to [21]
S9

NY climate network

Global Diversity Value (1.211324)	
10 Most Diverse Nodes	Node Diversity Value
77411	1.709796
31679	1.709502
30745	1.709361
72515	1.708603
30357	1.706790
39843	1.706774
87304	1.704423
71052	1.704219
98470	1.704098
83809	1.704047

TABLE S8: Human Genome: global and node diversity values of the 10 most diverse nodes in the NY climate network multiplex network.

London Transportation Network

Global Diversity Value (0.9455894)	
10 Most Diverse Nodes	Node Diversity Value
shepherdsbush	1.538358
kensington(olympia)	1.538358
westbrompton	1.538358
euston	1.538358
highbury&islington	1.538358
westhampstead	1.538358
blackhorseroad	1.538358
canadawater	1.538358
barking	1.538358
whitechapel	1.538358

TABLE S9: Human Genome: global and node diversity values of the 10 most diverse nodes in the London Transportation Network multiplex network.

* martin.ravetti@dep.ufmg.br

[1] A., S. & S., F. K. A distance measure between attributed relational graphs for pattern recognition. *IEEE Transactions on Systems, Man, and Cybernetics* **13**, 353–363 (1983).

[2] Zeng, Z., Tung, A. K. H., Wang, J., Feng, J. & Zhou, L. Comparing stars: On approximating graph edit distance. *Proc. VLDB Endow.* **2**, 25–36 (2009).

[3] Gao, X., Xiao, B., Tao, D. & Li, X. A survey of graph edit distance. *Pattern Anal. Appl.* **13** (2010).

[4] Lamberti, P., Majtey, A., Borras, A., Casas, M. & Plastino, A. Metric character of th equantum jensen-shannon divergence. *Physical Review A* **77**, 052311 (2008).

[5] Briet, J. & Harremoes, P. Properties of classical and quantum jensen-shannon divergence. *Phys. Rev. A* **79**, 052311 (2009).

[6] De Domenico, M., Nicosia, V., Arenas, A. & Latora, V. Structural reducibility of multilayer networks. *Nature Communications* **6** (2015).

[7] Nicosia, V. & Latora, V. Measuring and modeling correlations in multiplex networks. *Phys. Rev. E* **92**, 032805 (2015).

[8] Battiston, F., Nicosia, V. & Latora, V. Structural measures for multiplex networks. *Phys. Rev. E* **89**, 032804 (2014).

[9] Lacasa, L., Nicosia, V. & Latora, V. Network structure of multivariate time series. *Scientific Reports* **15**, 15508 (2015).

[10] Orsini, C. *et al.* Quantifying randomness in real networks. *Nature Communications* **6**, 8627 (2015).

[11] Schieber, T. A. *et al.* Quantification of network structural dissimilarities. *Nature Communications* **8**, 13928 EP – (2017).

[12] `https://github.com/tischieber/assessing_diversity_in_multiplex_networks/`.

[13] Sierra, S., Kupfer, B. & Kaiser, R. Basics of the virology of hiv-1 and its replication. *Journal of Clinical Virology* **34**, 233–44 (2005).

[14] Simon, V., Ho, D. D. & Karim, Q. A. Hiv/aids epidemiology, pathogenesis, prevention, and treatment. *Lancet* **9534**, 489–504 (2006).

[15] Levy, J. Hiv pathogenesis: 25 years of progress and persistent challenges. *AIDS* **23**, 147–60 (2009).

[16] S., M., T.W., C. & A.S., F. Pathogenic mechanisms of hiv disease. *Annual Review of Pathology* **6**, 223–48 (2011).

[17] Naif, H. Pathogenesis of hiv infection. *Infectious Disease Reports.* **5(Suppl 1)** (2013).

[18] Domenico, M. D., Lancichinetti, A., Arenas, A. & Rosvall, M. Identifying modular flows on multilayer networks reveals highly overlapping organization in interconnected systems. *Physical Review X* **5** (2015).

[19] Domenico, M. D., Porter, M. A. & Arenas, A. Muxviz: A tool for multilayer analysis and visualization of networks. *Journal of Complex Networks* **2**, 159–176 (2015).

[20] Stark, C. *et al.* Biogrid: a general repository for interaction datasets. *Nucleic Acids Research* **34**, D535–D539 (2006).

[21] De Domenico, M., Solé-Ribalta, A., Gómez, S. & Arenas, A. Navigability of interconnected networks under random failures. *Proceedings of the National Academy of Sciences* **111**, 8351 (2014).
